# Consumer Perception of Red Wine by the Degree of Familiarity Using Consumer-Based Methodology

**DOI:** 10.3390/foods10040749

**Published:** 2021-04-01

**Authors:** Jiyun Yang, Jeehyun Lee

**Affiliations:** Department of Food Science and Nutrition & Kimchi Research Institute, Pusan National University, Busan 46241, Korea; jiyunyang@pusan.ac.kr

**Keywords:** red wine perception, consumer familiarity, check-all-that-apply test (CATA), rating, consumer perception, red wine acceptability

## Abstract

Capturing and understanding consumers’ perceptions is not a simple quest, particularly for wine, which is one of the most complex beverages. In contrast to the increasing amount of wine import and consumption, studies on how Korean consumers perceive wine characteristics are limited. In this study, two different consumer-based questionnaires, check-all-that-apply (CATA) and rating, were used to compare the discrimination ability of samples and attributes. Consumer data were analyzed and compared to investigate whether the difference in the degree of familiarity with consumption frequency affects wine perception and preference. Consumers discriminated samples and attributes by sample using both scales, CATA and rating. It was confirmed that the CATA citation frequency reflected the rated intensity of the attributes in this study. Consumers who checked or did not check the CATA response rated the intensity of attributes differently. Different consumer subgroups based on familiarity also discriminated the samples effectively. However, users had a higher configuration similarity between the two questionnaires than non-users. Furthermore, the preference for wine might be affected by the degree of familiarity.

## 1. Introduction

Consumer perceptions of food products are difficult to define. Food perception is driven by a variety of factors, including sensory factors such as color, taste, and smell [1,2,3], and others such as expectations [4] or cognitive strategies [5]. Person-related factors, including physiological, psychological, biological, and even socio-cultural variables [6,7], may also affect product perception. Although wine perception is usually related to its intrinsic quality [8], consumer perception is dynamic, complex, and sometimes presents differences between what they perceive and their reaction [9]. To reveal the perceptible sensory attributes of foods and beverages, conventional techniques such as descriptive analysis have been carried out [10]. Currently, consumer-driven evaluations are also actively conducted to obtain direct information using consumer vocabulary, which is generally more understandable than terms used by trained panels [11].

Among the various factors influencing consumers’ perceptions, familiarity is one of the major factors. Product familiarity was explained as the evaluated judgment of consumers according to their subjective knowledge associated with the product [12,13]. Familiarity is influenced by the degree of previous exposure to the focal product [14] and affects acceptability and preference [15]. Consumers can easily detect and accept relevant product characteristics when they consume familiar products [16], but it may be more difficult for unfamiliar products [17]. Generally, consumers are unwilling to have unfamiliar foods due to the lack of information and understanding of the product [18], which may lead to lower consumption intentions by deriving less expectations [19]. The taste experience of consumers, as well as their peers, influences consumer preferences for food products [20]. Integrating consumption experience into food preference research might enable the observation of the true perception of consumers [21,22].

Wine, an alcoholic beverage often produced by the fermentation of *Vitis vinifera* [23,24], is one of the most researched and mentioned beverages in the literature [25,26]. This might be because of the complexity of wine characteristics [27] of unwontedness by varying brand, style, type, or even price [28,29]. In particular, wine is a product with a combination of several sensory characteristics and multidimensional aspects such as color, aroma, flavor, and mouthfeel [30,31]. For this reason, many wine sensory studies have been conducted to understand how consumers have perceived and understood wine in recent decades [27,32,33]. Red wine, with a wide sensory diversity, might be affected by consumers’ familiarity with culture, experience, knowledge, or exposure [28,34]. Wine consumption is increasing globally, and the Korean market has also continued to increase in imports and consumption [35,36]. Few studies have been conducted on sensory scientific research into the wine perception of Korean consumers, in contrast to the increasing trend of wine consumption.

To understand how consumers perceive product characteristics, various techniques are used by sensory scientists. Among the numerous methodologies, novel and quick methods have gained interest in recent years [37]. Check-all-that-apply (CATA) is regarded as a prominent approach to describe and differentiate samples based on their attributes perceived by consumers [38]. CATA questions are composed of predefined sensory descriptors, and participants select all the terms to describe samples appropriately [39,40]. This method has some advantages, such as simplicity, ease, and quick response time [41]. In addition, the data from CATA are considered valid and repeatable [42,43,44]. However, the binary response of CATA has a limitation in that it does not allow the measurement of the intensity of the attribute [45]. This limitation led to the application of intensities such as CATA with intensity [46] or rate-all-that-apply (RATA) [47,48,49]. In particular, RATA is considered to have the potential for intensity-based variants of CATA [46], although it is controversial because of statistical analysis difficulties [45,50].

Some studies have been conducted on consumer perceptions of wine, including both intrinsic and extrinsic perceptions, using consumer-based methods including CATA or RATA [39,47,51,52,53]. These studies focused on how consumers characterized a specific group of wine samples or compared performance ability between methods. Research on consumer acceptability of wine has focused on sensory drivers affecting preference [28,29,54,55]. However, few studies have been conducted to advance the understanding of how consumer perception of wine differs in their familiarity with wine, not the degree of knowledge, expertise, or education.

The objectives of this study were: (i) to investigate consumer perceptions of wine with a complex flavor, taking into account the degree of familiarity; and (ii) to determine which sensory method is more suitable for capturing consumer perceptions of wine. The consumer acceptability of wine was also evaluated to compare differences by consumer group. Three criteria were considered to gain deeper insight into consumers’ perception of wine and their ability to undertake the evaluation task: (1) to identify differences between two questionnaires, CATA vs. rating, by focusing on discrimination ability, relations between CATA frequency and rated intensity, and sample configuration; (2) to investigate the relationship between perception and familiarity of wine by comparing acceptability and task performance ability of consumer subgroups by familiarity; and (3) to find consumer segmentation by the acceptability of wine.

## 2. Materials and Methods

### 2.1. Samples

Preliminary tests were conducted to select samples that were used in the consumer test. In the first stage, twenty-two commercial red wines, reflecting market share and mindshare in Korea, were considered and purchased. Then, researchers selected six samples with different flavor, origins, and cultivars after tasting by consensus. Detailed sample information is shown in Table 1, including product name, type, cultivar, vintage, country, alcohol content, and price. All wine samples were purchased at a department store or wine shop and stored at 15 °C in a wine refrigerator (LG Dios W715B, LG Electronics, Changwon, Korea).

### 2.2. Participants

A total of 122 consumers (male = 52, female = 70, aged between 19 and 65 years) were recruited from the community of Pusan National University through an online screening survey (SurveyMonkey, Palo Alto, CA, USA), after approval by the Institutional Review Board (IRB) of Pusan National University (PNU IRB/2018_23_HR). Consumers who had no food allergies and could drink alcohol beverages were selected. All participants signed a consent form to confirm their voluntary participation and were given compensation.

### 2.3. Consumer Test

**Figure 1:**
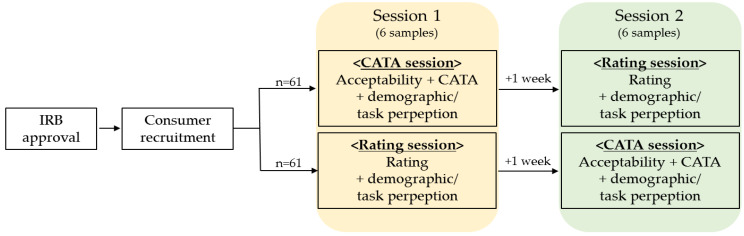
Flowchart of wine perception consumer test.

In this study, consumers participated twice (with a week in between sessions) and evaluated samples using the check-all-that-apply (CATA) and rating (0–5 point scale) questionnaires. In the case of the rating scale, a 0-point was added to serve as a “no intensity perceived” category so that ratings could be investigated in relation to how consumers use CATA. Half of the consumers evaluated samples using the CATA questionnaire in the first session and the rating questionnaire in the second session, and the rest evaluated them conversely. Each questionnaire was composed of 108 sensory terms (Table 2), which were selected based on the Wine Aroma Wheel developed by researchers at UC Davis [56]. Other modalities were not evaluated to keep consumers focused on their evaluation of wine aromatics from retro-nasal olfaction. Attribute reduction was not conducted to investigate how general consumers perceive wine and use already developed terminologies. The order of terms was presented in a fixed sequence by the Korean alphabet to help consumers utilize a long list of terms to evaluate samples more easily [57]. The overall acceptability of each sample was assessed before the CATA task using a 9-point hedonic scale. About seven minutes were given per sample to allow enough time for evaluation, including a break. Additional time was provided for consumers who needed more time. At the end of each evaluation, demographic and task-perception questions were asked of the consumers. The task perception question included “the degree of ease of answering the CATA/rating questionnaire” and “the degree of tediousness” to answer the CATA/rating questionnaire to compare consumers’ perceptions of CATA and rating [58] using the 5-point Likert scales. A schematic flowchart of consumer evaluation is shown in Figure 1.

Each sample (30 mL) was served in a Riedel “O” red wine glass (Cabernet/Merlot, Riedel, Kufstein, Austria) for evaluation with a 3-digit random code and presented monadically following Williams’ Latin Square design [59]. All samples were prepared immediately before serving and served at room temperature (21 °C). Whole wheat crackers (Integrali ricchi in fibre, Nuova Industria Biscotti Crich S.p.a., Regione del Veneto, Italy) and bottled water (Samdasoo 500 mL, Kwang Dong Pharmaceutical. Co., Seoul, Korea) were provided as palate cleansers. An empty cup was given, and consumers could expectorate wine samples after evaluation to prevent fatigue from alcohol absorption.

All tests were conducted in the evening after 6 p.m. in the sensory booth at Pusan National University. As a precaution, participants had to rest for approximately 30 min. after finishing all the evaluations to minimize any possible problems caused by alcohol consumption.

### 2.4. Data Analysis

The CATA binary data were converted into the selection frequency of terminology, and data were analyzed using Cochran’s Q test [60] to determine significant differences between samples by each term. Two-way analysis of variance (ANOVA) with wine as the fixed factor and consumers as the random factor was conducted to analyze the rating data of attribute intensity and acceptability of wine samples. To visualize and simplify the relative position between samples and their characteristics, correspondence analysis (CA) using CATA data and principal component analysis (PCA) using rating data were conducted. The RV coefficient test [61] was performed to determine the similarity of sample evaluation between the two methods by comparing sample configurations resulting from CATA and rating. Linear regression was used to confirm whether the CATA term citation frequency reflects consumers’ perceived intensities. Collected data from participants were used for statistical analysis as total consumers, and we also compared the sub-groups differing in familiarity with wine as users and non-users. For the subgroups, the same statistical analyses, including Cochran’s Q test, CA, ANOVA, PCA, and RV coefficient were conducted. Cluster analysis using Ward’s method was also conducted to segment consumers according to their wine sample acceptability. All statistical analyses were performed at a significance level of 0.05 (α = 0.05).

Two-way ANOVA, CA, PCA, and cluster analysis were conducted using SAS^®^ software 9.4 (SAS Institute Inc., Cary, NC, USA). Cochran’s Q test, linear regression, and RV coefficient analyses were carried out using the XLStat^®^ software package (version 2020.2.1., Addinsoft SARL, New York, NY, USA).

## 3. Results

### 3.1. Comparison of Consumers’ Wine Perception by CATA and Rating Scales

#### 3.1.1. Significant Term and Its Number in CATA vs. Rating

Table 2 shows the terminology of wine used in both evaluations, CATA and rating. One-hundred and eight terms were divided into three groups by the selected frequency of CATA task. The criteria for dividing groups were based on previous studies [57,62,63], which ranged from 10% of the frequency in at least one sample to a 20% cut-off point during emotion term development in the CATA task [63]. Only 11 descriptors were selected by more than 10% of participants for at least one sample.

In order to compare consumers’ wine perceptions measured using the two methods, the significance of the terms used in each questionnaire was analyzed. Both the CATA and rating methods discriminated samples based on wine characteristics, but a different tendency was observed. Table 2 shows a summary of the terms with significance for CATA and rating by all consumers. Among the 108 descriptors of wine flavor, 14 terms for CATA and 18 terms for rating showed significant effects for describing six red wine samples. Only two descriptors, *honey* and *raisin*, showed significance for both methods, which might be evidence that consumers evaluated wine differently when using CATA and rating questionnaires.

**Figure 2:**
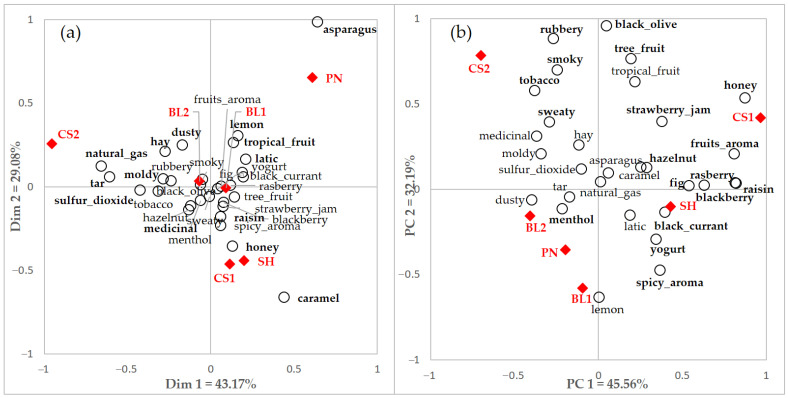
(**a**) Correspondence analysis (CA) and (**b**) principal component analysis (PCA) of total consumer data with integrated significant terms (*n* = 30) by CATA and rating of total consumers. Filled diamonds indicate a red wine sample and empty circles indicate an attribute. Terms in bold indicate significant differences shown among samples.

Figure 2 represents the CA and PCA biplots using CATA and rating data of total consumer responses using significant terms for CATA and rating, respectively. Samples were discriminated effectively by their sensory attributes, but some similarities and dissimilarities were observed. The X-axis of these two biplots showed similar descriptions of the properties. Fruit or sweet related attributes such as *caramel*, *honey*, and *raisin* were positioned on the right side of the X-axis, and some characteristics regarded as negative wine attributes such as *dusty*, *natural gas*, and *tar* were located on the left side. However, the sensory characteristics appeared more spread on the PCA plot than the CA plot.

**Figure 3:**
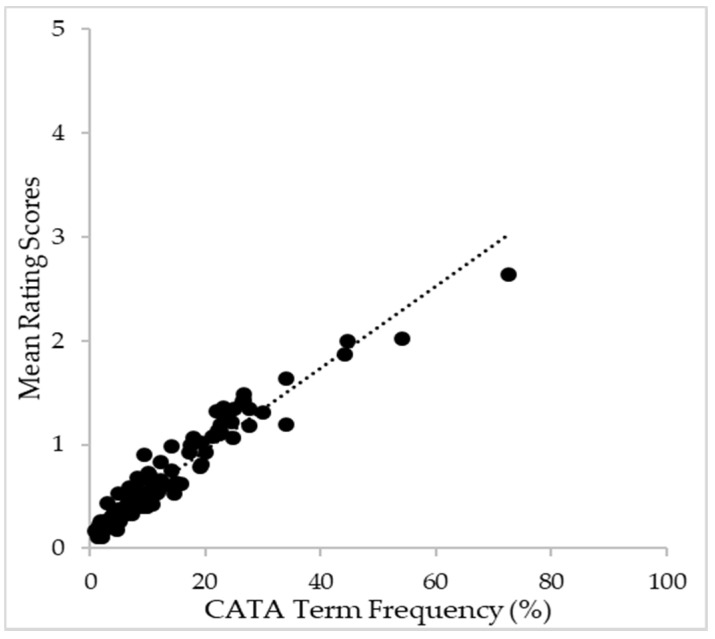
Plot of relation between CATA citation frequency (0–100%) according to mean rating score (0–5 point scale) using all sensory terms provided to consumers.

To address the relationship between the CATA citation frequency and the rated intensity of attributes [57], linear regression analysis was conducted [45,54]. Figure 3 shows that consumer results indicate a strong relationship between CATA and rating for red wine perception. Plots indicate that as the use of the CATA term frequency increases, the rating score also increases linearly with the linear index R^2^ = 0.929.

#### 3.1.2. Checked or Not Checked in CATA and Their Respective Rating Intensity Comparisons

To obtain more information about the relationship between CATA and rating, checked or not checked in the CATA response and their respective intensity by rating were compared for the six most frequently checked attributes in CATA, including *alcohol*, *artificial fruit*, *ethanol*, *fruit aroma*, *oxidized*, and *pungent*. The most frequently selected terms could be regarded as having a high intensity of these attributes. As shown in Table 3, the frequency of the CATA term and the mean rating intensity were analyzed using six red wine samples for the six most selected attributes. In addition, a *t*-test was performed to determine whether there was a difference in the rating intensity of the people who selected and did not select the corresponding characteristic in CATA.

When comparing rating means by total consumers, only one attribute, *fruit aroma,* showed a significant difference among the six samples. Despite the high frequency of CATA selection, these six terms did not show significant differences between samples in the Cochran’ s Q test using CATA responses. Different results were found for each characteristic when comparing the rated attribute intensities of those who checked or did not check for characteristics. Overall, the participants who checked the terminology in CATA showed higher ratings of response intensity. In every attribute, more than half of the six wine samples showed significantly different intensities between the two groups. Among them, *ethanol* and *oxidized* showed significant differences among all wine samples. Although two terms, *alcohol* and *fruit aroma,* showed less discrimination ability of the sample between the two groups by CATA response, these terms had significant differences for half of the six samples.

#### 3.1.3. Wine Sample Configuration Comparison between CATA and Rating

The RV coefficient was calculated to investigate how similarly consumers evaluated samples using the CATA and rating questionnaires. The first two dimensions of the sample aspect of CA and PCA were used for the CATA and rating data, respectively. The RV coefficient value between CATA and the rating method was a moderate value of 0.636 (*p* = 0.099).

### 3.2. Effects of Familiarity with Wine

To investigate consumer perception difference by familiarity, consumers were divided into two groups based on their consumption frequency, as users and non-users. Consumers who drink wine more than once a month were regarded as users. The detailed consumption frequency information is shown in Table 4.

#### 3.2.1. Comparison of Significant Terms between Users and Non-Users of Wine Evaluation Using CATA

Cochran’s Q test was conducted to determine attributes showing significant differences among samples, and their numbers were compared between user and non-user groups to investigate whether sample familiarity affects wine sensory characterization using CATA questions (Table 5). When simply comparing the number of terms, there was a difference between users and non-users. Users had 12 terms with significant differences between samples, whereas non-users had only four attributes that showed significant differences. The number of significant descriptors for all consumers was 14, which is similar to that of the users. Overall, consumers who were familiar with wine could discriminate samples using the CATA questionnaire.

In addition to the differences in the number of significant terms, users and non-users had only a few common terms. Between users and non-users, only one term, *sulfur dioxide,* overlapped, which might suggest that consumers performed sensory characterization of red wine differently based on their familiarity with wine. Another difference between the two groups was that users discriminated more attributes that could be regarded as negative, such as *burnt toast*, *hay*, *medicinal moldy*, *rubbery*, and *tar,* than non-users. Detailed data are not shown, but it should be noted that terms with significant differences did not have a high selection frequency for each sample. Additionally, eight terms (*asparagus*, *hay*, *lemon*, *medicinal*, *moldy*, *natural gas*, *tar*, and *tropical fruit*) were significant in users only and all consumers, and only two terms, *honey* and *raisin*, commonly showed significance between non-users and all consumers. The term *sulfur dioxide* was the only term that showed significance for all consumers, users, and non-users.

**Figure 4:**
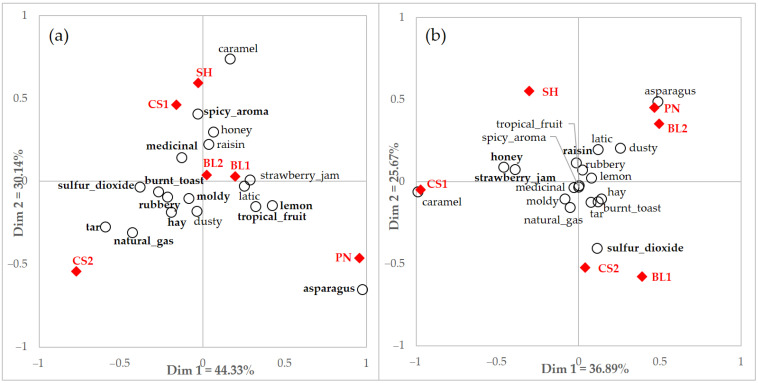
Correspondence analysis (CA) of (**a**) users and (**b**) non-users data with integrated significant terms (*n* = 18) by CATA questionnaire of total consumers, users, and non-users. Filled diamonds indicate red wine samples and empty circles indicate attributes. Terms in bold indicate significant differences according to each consumer group with different familiarity.

A CA biplot using CATA data with significant terms by users and non-users is shown in Figure 4. Both groups discriminated the samples based on their perceived attributes. Consumers perceived some samples similarly, such as sample PN with *asparagus*, CS1 with *caramel*, and CS2 with *sulfur dioxide*. However, differences were also represented by the sample location on the CA biplot.

#### 3.2.2. Significant Terms Comparison between Users and Non-Users of Wine Evaluated Using Rating

The sensory characterization of red wine using the rating questionnaire was different from the CATA method. When comparing significant terms from ANOVA and its number, a similar discrimination ability was observed between the user and non-user groups. Nine descriptors showed significant differences for users and 10 attributes for non-users.

However, there was no common descriptor between the two consumer groups, which means that they evaluated samples differently using terms from the rating method. On the other hand, there were several common terms when comparing the two groups with all consumers. Eight of the nine significant descriptors of users overlapped with all consumers, including *black olive*, *honey*, *rubbery*, *smoky*, *spicy aroma*, *sweaty*, and *tobacco*. On the other hand, the non-user group used three terms similar to all consumers, such as *fig*, *fruit aroma*, and *strawberry jam*.

**Figure 5:**
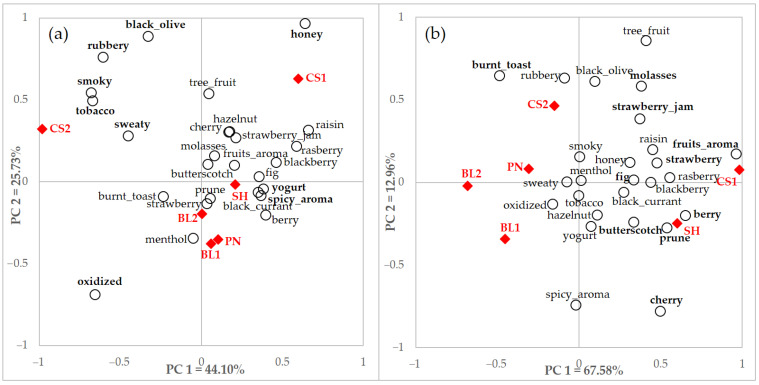
Principal component analysis (PCA) of (**a**) users and (**b**) non-users data with integrated significant terms (*n* = 26) by rating questionnaire of total consumers, users, and non-users. Filled diamonds indicate red wine samples and empty circles indicate attributes. Terms in bold indicate significant differences according to each consumer group with different familiarity.

A PCA biplot using rating data with significant terms by users and non-users is shown in Figure 5. Both groups also discriminated the samples based on their perceived characteristics. There is a similar explanation for PC 1 as fruit and berry-related attributes were located on the right side and negative characteristics were located on the opposite side. Sample CS1 with berry attributes and CS2 with relatively negative attributes were also similar to the results of the PCA by users and non-users. Although differences were also shown, such as sample location, users and non-users discriminated samples effectively by perceived attributes using a rating scale.

#### 3.2.3. Wine Sample Configuration Comparison between CATA and Rating by User and Non-User Consumers

The RV coefficient was calculated to understand whether the familiarity with the sample affects the sample configuration between the CATA and rating methods. The RV coefficient of the sample configuration by users was 0.786 (*p* = 0.017) and 0.382 (*p*-value = 0.340) for non-users. A much lower RV coefficient with a high *p*-value by non-users than by users shows that participants used CATA and rating methods differently. This also means that consumers’ familiarity with the sample could affect wine characterization using different questionnaires.

#### 3.2.4. Acceptability of Wine

Significant differences in liking existed among samples, which meant consumers evaluated the acceptance of six wine samples differently, reflecting their preference. In general, consumers liked all samples moderately, and the mean acceptability score ranged from 4.5 to 5.4. Participants liked the SH sample the most and CS2 the least. Liking scores of the two groups differing in familiarity were also analyzed. The acceptability of users tended to be slightly higher than that of non-users. For users, four of six samples got more than five points, which would be between ‘neither like nor dislike’ and ‘like slightly’. The liking tendency of users seems similar to the acceptability of all consumers. Non-users evaluated their liking negatively for five of the six samples, and only the SH sample was rated higher than five points.

### 3.3. Consumers’ Acceptability Clusters

Cluster analysis was performed using consumers’ liking scores, and there were four clusters with differing acceptability. The number of clusters was determined by dendrogram and reflecting how to discriminate consumer preference patterns meaningfully. As shown in Table 6, most consumers in cluster 1 are considered as ‘neutral’ likers. They showed a liking score of around five points for all samples, which means they did not show a clear preference for any sample. They liked the SH sample the most and CS2 the least. The second largest consumer subgroup was cluster 2, which included consumers who generally disliked samples and could be regarded as ‘dislikers.’ They showed a liking score lower than the neutral five points (mean scores ranged from 3.3 for CS2 to 4.4 for CS1) for all red wine samples. Cluster 3 consisted of acceptors for wine samples except sample BL1, but the number of consumers was only 11, the smallest of all clusters. Sample CS1 had the highest liking, and BL1 had the lowest score. Cluster 4 showed a different tendency from the other clusters, and respondents evaluated their liking distinctly for the six samples. Unlike other subgroups, the difference between the highest and lowest acceptability was over three points out of the nine-point scale. The least liked sample was BL2 (3.0), and the most liked sample was PN (6.7).

## 4. Discussion

### 4.1. Investigation of Consumers’ Wine Perception Using Consumer-Based Methodologies

To understand how consumers perceived red wine, consumer-based techniques such as CATA and rating methods were used to collect data and compare the results. Similar consumer studies have compared CATA and other intensity methods, such as RATA [49] or CATA with intensity [46]. The results from these consumer-based methodologies had similarities and/or dissimilarities, but no one method could be considered superior to the others.

The sample discrimination ability between CATA and rating could be compared with the number of terms with significant differences. In this study, 14 terms for CATA and 18 terms for rating showed significant differences, indicating that the rating techniques showed slightly higher discrimination ability. However, the degree of this difference might deserve further consideration as a total of 108 attributes were included in the terminology list. Because more than one hundred terms might be difficult for consumers to evaluate, to alleviate this, the terms were presented in Korean alphabetical order [57]. The use of fixed CATA terms requires less time and provides more cognitive capacity for the evaluation [64,65,66]. In fact, consumers did not perceive either the CATA or the rating tasks as boring or difficult when asked about task perception (Table 4).

These results, in the case of significant terms in CATA and/or rating, were visualized through CA and PCA, respectively, because it is difficult to identify the differences in wine perception by simply comparing the number of significantly different terms. Samples were discriminated effectively regardless of the scales used for the CA and PCA, even though some differences existed. A similar result was reported in a study comparing CATA and RATA [48,49]; RATA was associated with the same or higher percentage of terms with significant differences compared to CATA. However, the number of terms was less than in the current study, and RATA and rating methods are different tasks.

When comparing the relationship between the CATA term citation frequency and rated attribute intensity in this study, a near-linear (R^2^ = 0.929) relationship was confirmed. This result indicates that the CATA frequency potentially reflects the attribute intensity when Korean consumers evaluate red wine perception. This is in line with the findings of other researchers [48,58] using various food categories. Their results agreed with ours that the frequency of CATA indirectly implies each attribute’s intensity. Nevertheless, this analysis alone could not advance the understanding of the relationship between CATA as a binary response and rating as an intensity-based response. Further investigation on how the CATA term is chosen would be beneficial to understand consumers’ perception of red wine with complexity using different questionnaires.

To understand the consumers who did not check for CATA questions even though they perceived particular attributes, their respective ratings were compared. The mean attribute intensity was not zero for consumers that did not check the CATA question. When checked and not checked were compared for the six most selected terms, there were significant differences in three of the six samples, and significance in all samples for two terms. These results imply that there is a difference in perceived intensity between the groups that checked and did not check by CATA. This might be regarded as the presence of an individual-specific threshold, which means that consumers do not check all the attributes they perceive, but only those that are more intense than their internal threshold [67].

The rating method used in this study might supplement the limitation of RATA or CATA variants with the intensity method by collecting the intensity of all attributes. A well-known consumer-based rating method, such as RATA, asks the consumers to rate the intensity only of attributes applicable to the focal sample. In such cases, the statistical analysis of unselected terms or missing data can be difficult. Therefore, the response to the unselected attribute is replaced with a zero for analysis [45,50]. However, since this is hardly seen as a complete response from the consumer, the rating method might be considered to have better statistical power. Moreover, because the rating method was used in this study, it was possible to directly compare the intensity of each attribute according to the CATA response.

The RV coefficient implies how consumers characterized wine samples similarly or differently using the CATA and rating methods. Moderate sample configuration similarity between the two methods was confirmed by the RV coefficient (0.636), and this result suggests that consumers used CATA and rated somewhat differently. This sample configuration comparison using the RV coefficient was lower than other comparative studies between methods (such as 0.90–0.97 for CATA, CATA with intensity and Napping^®^ using eight different beers [46], 0.82–0.97 for CATA and RATA by four different product categories [49], and 0.81–0.99 for four of the six studies between CATA and RATA by different consumer studies [48]). Relatively low RV values, between 0.61 to 0.80, were also reported in the study of Vidal et al. [48]. Vidal et al. [48] mentioned that the low RV coefficient between sample configurations indicated potential differences regarding similarities and differences among samples between methods. However, the relatively low RV value in this study might be due to the large number of characteristics or the consumer’s unfamiliarity with the sample.

Identical terminologies of wine aromatics were used, and the same consumer participated in the evaluation of red wine using both the CATA and rating questionnaires in this study; thus, direct comparison was possible. However, the terms used for flavor evaluation were based on the developed wine aroma wheel, and taste attributes such as sweetness, saltiness, bitterness, sourness, and trigeminal perception astringency were not included. As bitterness, astringency, and sweetness are very important and critical characteristics of wine, additional information on consumer perception would be provided if these basic taste terms were added in future studies. When evaluation is restricted into one sensory modality from what respondents perceived, a “dumping effect” may occur and their perception and rating could be expressed using other descriptors [68].

### 4.2. Comparison of Wine Perception Differences for Consumer Group According to Familiarity

In order to determine whether the difference in consumer perception of wine was related to consumers’ familiarity with wines in general, the results were analyzed and compared according to the consumer’s familiarity with wine as users and non-users, determined by consumption frequency. Prior studies have suggested that consumers’ familiarity, consumption period/frequency, and exposure to the product strongly influence flavor perception and/or preference [55,69,70].

To identify whether there was a difference in attribute perception according to the familiarity of the product, consumer groups differing in familiarity were compared for each method. When comparing discrimination ability using CATA between users and non-users by the number of significant terms, users showed a better ability to discriminate attributes between samples. However, similar discrimination ability by the rating method resulted between the two groups, although non-users had one more term with significance. This could be an indication that users showed better discriminating performance when using CATA, but non-users used the rating method more efficiently. However, more consideration is needed when interpreting the results. Although the number of significant terms was different and different terms were used to characterize samples when comparing results from the CA and the PCA, consumers with different familiarities showed similar discrimination of samples. In the study by Vidal et al. [48], consumers used more terms to describe samples when using RATA compared to the CATA questionnaire. However, different discrimination abilities were obtained depending on how RATA data were analyzed, such as RATA-as-CATA or its rated score. This may suggest the importance of correctly interpreting data because the result could be interpreted differently depending on the direction of the analysis, even when the same data are used.

Interesting results have been observed in the use of terminology according to the evaluation method. Similar to other reported literature [46,48], consumers in the current study tended to use terms that are not commonly used when evaluating with CATA and used relatively familiar descriptors when conducting the rating method. For example, *sulfur dioxide* is not a common word for general consumers. However, it was significantly used for discriminating samples by all consumers, users, and even non-users. This might be related with the fact that CATA showed more discrimination ability when evaluating minor, low intensity, less simple, or novel attributes, and RATA was considered more acceptable for evaluating samples with similar characteristics but different intensities [46,48].

However, a distinctively superior ability to discriminate between two consumer groups when using CATA or rating questions was not shown in this study. Only different tendencies to evaluate wine samples using different questionnaires were seen. This is in line with other studies that have compared CATA and RATA [48], CATA and CATA with intensity [46], or RATA and descriptive analysis [47]. In these studies, no identical results, including descriptive or discriminative ability, were found between the compared methods. However, a similar tendency to discriminate the sample was shown. Similarly, consumers were able to classify samples according to their characteristics regardless of the evaluation method used. When considering the RV coefficient, comparing configurations between users and non-users using CATA and rating scales, high RV values were obtained by users. This indicates that users who are familiar with red wine might perceive and evaluate samples similarly regardless of the scale used.

The samples used in this study were liked differently by the two consumer groups. Users liked wine samples slightly more than non-users, and this might be affected by the familiarity of wine. Product familiarity plays an important role in consumers’ preferences and acceptability by generating a better match between the sensory attributes of a product and consumers’ expectations [71,72]. It is also adapted to different cultural consumer groups [15]. Expectation of the wine sample might also affect acceptance by consumers. Users might know and understand the basic attributes of wine, and even some characteristics such as earthy, yeast, or vegetative aroma, which negatively correlate with consumers [54]. However, these attributes might affect non-users and decrease their liking. The CS2 sample had the lowest liking score by both the users and non-users groups, which was related to *sulfur dioxide*, *tar*, and *rubbery* attributes in the CA and PCA plots (Figure 2, Figure 4 and Figure 5). *Sulfur dioxide* and *smoky* characteristics might contribute to reducing the acceptance of wine [29,54,55]. *Tar* and *rubbery* attributes could also be regarded as disliking drivers in this study. Wine samples were selected to include flavor ranges among different types, cultivars, producing country, and price range. Wine faults were not considered as exclusion criteria.

### 4.3. Consumer Acceptance of Wine by Their Preference Trend

Cluster analysis of wine samples revealed different clusters based on consumer preferences, which could be defined as neutral likers, dislikers [54], acceptors, and discriminators. These four clusters have distinctively different preferences. There were no commonly liked or disliked samples between the clusters. Based on acceptability, cluster 1 was regarded as neutral likers and cluster 2 as dislikers, and both groups were non-discriminators who rated their liking for all samples similarly; the mean range between the most and least liked one was about one point. In contrast, cluster 3 was an acceptor and cluster 4 was a discriminator, and both showed relatively large intervals, such as 2.5 points for cluster 3 and 3.7 points for cluster 4.

Similar liking tendencies of wine samples between clusters were also shown in other studies [29,54]. In the study by Biasoto et al. [54], five clusters existed, one of which was general likers (liking score from 5.51 to 7.30) and another cluster was categorized as dislikers (mean liking ranged from 1.90 to 4.08). The others were not classified according to the liking tendency. Consumers who evaluated Australian Cabernet Sauvignon and Shiraz wine were divided into four clusters according to their acceptability: two clusters generally liked all samples and gave over six points on the nine-point scale, and the other two clusters had a wide range of liking, with several samples rated less than five [29]. These results indicated that not all consumers had the same liking, but had different preferences according to their own standards.

When comparing sample preferences according to product familiarity, CS2 was generally disliked, and CS1 and SH were liked. However, this preference was not observed for all clusters. Sample CS2 received the lowest liking score by cluster 1 and cluster 2, but it was rated the second highest score by cluster 3. In the case of sample BL2, which was moderately liked by total consumers and consumer subgroups by familiarity, it had significantly low acceptability by cluster 4. The BL2 sample was related to dusty and asparagus attributes as evaluated by non-users using CATA (Figure 4), which might have affected cluster 4 as discriminators, which were mostly composed of users. Green flavors related to vegetables were negative drivers for consumer liking [28,29], and *asparagus* might be regarded as a green flavor in this study.

Considering the consumer ratio by familiarity of each cluster, more than 72% of clusters 3 and 4 were users, which might contribute to liking tendency. Furthermore, a positive correlation between users and cluster 4 (*r* = 0.855, *p* = 0.03) was observed when comparing the correlation between each group by familiarity and each cluster using the Pearson correlation coefficient. These results also support that product familiarity related to consumption frequency affects consumers’ perceptions and preferences for red wine. Further analysis might be difficult because the number of consumers in clusters 3 and 4 is too small, but it is expected that more meaningful results would be derived if consumer perception was evaluated using acceptors and discriminators in future studies.

## 5. Conclusions

The purpose of this study was to understand how consumers perceive red wine, which is regarded as a complex alcoholic beverage, considering various factors, questionnaire methods (CATA and rating scales), familiarity with red wines, and consumer segmentation based on their preferences.

Consumers used CATA and rating scales efficiently to evaluate red wine based on their perceived attributes. Among the 108 attributes, significant terms were limited and differed according to the questionnaires used and the wine familiarity of consumers. However, consumers discriminated samples similarly regardless of scales and familiarity, although the number of significant terms was different. Consumers tend to use novel terms when conducting CATA but use familiar terms when using the rating method. Similar to other comparison studies of CATA and intensity-based methods, it was confirmed that the CATA term citation frequency also reflects attribute intensity in this study. Even though there was no significant difference in the discrimination ability in performance, users might experience less difficulties than non-users when they evaluate samples. Furthermore, consumers have different preferences for segmentation. Among four consumer clusters by liking, only the acceptors group was positively related to consumers’ subgrouping by more familiar users.

There are several limitations to this study. The wine consumption frequency of the user group was not high compared to that in previous studies. Although Koreans’ wine intake has increased markedly, wine is not mainly consumed in alcoholic beverages, unlike in Western countries. Even though this study has confirmed that participants have the ability to discriminate samples using a large number of terms, more than 100 terminologies, including unfamiliar terms to general consumers, could have affected consumer evaluations. Further research with a reduced list of attributes and considering consumers with other alcohol beverage consumption would be needed to understand consumer perceptions of complex beverage categories such as wine. These results would be helpful not only to those who want to know consumer perceptions using consumer-based evaluation methods, but also those who want to use them as marketing sources based on consumer perceptions.

## Figures and Tables

**Table 1:** Sample information.

Label	Product Name	Type	Cultivar	Vintage	Country	Alcohol (In Label)	Price (KRW) ^1^	Price (USD)
BL1	Ca’Marcanda Promis	Blending	Merlot, Syrah, Sangiovese	2014	Italia	13%	65,000	57.52
BL2	Marqués de Riscal, Reserva	Blending	Tempranillo, Graciano, Mazuelo	2013	Spain	14%	40,000	35.40
CS1	Columbia Crest	Monovarietal	Cabernet Sauvignon	2016	USA	13.5%	20,000	17.70
CS2	Cono Sur Bicicleta	Monovarietal	Cabernet Sauvignon	2017	Chile	13.5%	14,500	12.83
PN	Chambolle–Musigny Louis Jadot	Monovarietal	Pinot Noir	2014	France	13%	130,000	115.04
SH	The Lackey	Monovarietal	Shiraz	2016	Australia	14.5%	19,000	16.81

^1^ The exchange rate of 1130 South Korean won (KRW) is approximately 1 United States dollar (USD) (as of November 2018).

**Table 2:** Terminology of 108 wine characteristics used in the check-all-that-apply (CATA) and rating questionnaires ^1^.

Group A ^2^	Group B ^3^				Group C ^4^		
**Dusty**	Acetic acid	Dried	Leesy	Rubbery	Almond	Diacetyl butter	Skunk
**Hay**	Alcohol	Dried fruit	Licorice	Sauerkraut	Artichoke	Eucalyptus	Strawberry jam
**Honey**	Apple	Earthy aromas	Menthol	Smoky	**Asparagus**	Garlic	Sulfur
**Lactic**	Apricot	Ethanol	Micro scents	Soybean	Bacon	Geranium	Sweaty
**Lemon**	Artificial fruit	Ethyl acetate	Mint	Spicy	Banana	Green beans	**Tar**
**Medicinal**	Berry	Fig	Moldy cork	Spicy aroma	Bell pepper	Hazelnut	Vanilla
**Moldy**	Black currant	Floral	Orange blossom	Strawberry	Black pepper	Hydrogen sulfide	Violet
**Natural gas**	Black olive	Floral aromas	Oxidized	Tea	Burnt match	Melon	Walnut
**Raisin**	Blackberry	Fresh	Petroleum	Tobacco	Butterscotch	Molasses	Wet wool
**Sulfur dioxide**	Burnt toast	Fruits aroma	Phenolic	Tree fruit	Cabbage	Mushroom	
**Tropical fruit**	Cedar	Grapefruit	Pineapple	Wood aromas	**Caramel**	Oak	
	Chemical	Green grass	Prune	Yeast	Chocolate	Other	
	Cherry	Green olive	Pungent	Yogurt	Cloves	Peach	
	Citrus	Herbaceous	Raspberry		Coffee	Plastic	
	Diesel	Kerosene	Rose		Cooked	Resinous	

^1^ Terms in bold showed significant differences between samples when tested with Cochran’s Q test using CATA data (*p* = 0.05). Underlined terms showed significant differences between samples when tested with ANOVA using rating data (*p* = 0.05). ^2^ Group A: terms selected by more than 10% of participants for at least one sample and showed significant differences among samples. ^3^ Group B: terms selected by more than 10% of participants for at least one sample but did not show significant differences among samples. ^4^ Group C: terms selected by less than 10% of participants for all samples.

**Table 3:** The mean results of intensity rating and summation of frequencies from the CATA questionnaire for the six most selected terms (*n* = 122) ^1^.

Wine Sample	Rating Mean ^2^	CATA Frequency Sum	Rating Mean		*t*-Value	*p*-Value
			Not check (*n*)	Check (*n*)		
Alcohol						
BL1	2.7	85	2.2 (*n* = 37)	2.9 (*n* = 85)	−2.73	0.0074
BL2	2.7	91	2.3 (*n* = 31)	2.8 (*n* = 91)	−1.89	0.0609
CS1	2.5	90	2.2 (*n* = 32)	2.6 (*n* = 90)	−1.44	0.1538
CS2	2.7	87	1.8 (*n* = 35)	3.1 (*n* = 87)	−4.79	<0.0001
PN	2.6	88	1.9 (*n* = 34)	2.8 (*n* = 88)	−3.60	0.0005
SH	2.6	91	2.3 (*n* = 31)	2.8 (*n* = 91)	−1.61	0.1103
Artificial fruit						
BL1	1.7	46	1.3 (*n* = 76)	2.4 (*n* = 76)	−4.60	<0.0001
BL2	1.6	36	1.4 (*n* = 86)	2.2 (*n* = 36)	−2.82	0.0056
CS1	1.6	35	1.5 (*n* = 87)	1.9 (*n* = 35)	−1.72	0.0875
CS2	1.6	44	1.2 (*n* = 78)	2.3 (*n* = 43)	−4.88	<0.0001
PN	1.6	41	1.2 (*n* = 81)	2.5 (*n* = 41)	−5.80	<0.0001
SH	1.7	47	1.4 (*n* = 75)	2.0 (*n* = 47)	−2.54	0.0123
Ethanol						
BL1	2.0	55	1.4 (*n* = 67)	2.7 (*n* = 55)	−4.86	<0.0001
BL2	1.9	58	1.4 (*n* = 64)	2.4 (*n* = 58)	−4.03	<0.0001
CS1	1.8	52	1.4 (*n* = 70)	2.4 (*n* = 52)	−3.92	0.0001
CS2	1.8	60	1.2 (*n* = 62)	2.6 (*n* = 60)	−5.55	<0.0001
PN	1.8	50	1.2 (*n* = 72)	2.6 (*n* = 50)	−6.17	<0.0001
SH	1.9	48	1.4 (*n* = 74)	2.6 (*n* = 48)	−4.77	<0.0001
Fruit aroma						
BL1	2.0 ^abc^	64	1.7 (*n* = 57)	2.3 (*n* = 64)	−2.32	0.0221
BL2	1.8 ^c^	65	1.5 (*n* = 57)	2.0 (*n* = 62)	−2.03	0.0451
CS1	2.2 ^a^	70	2.0 (*n* = 52)	2.4 (*n* = 70)	−1.67	0.0970
CS2	2.0 ^bc^	59	1.7 (*n* = 61)	2.2 (*n* = 59)	−1.57	0.1180
PN	2.1 ^ac^	71	1.9 (*n* = 51)	2.2 (*n* = 71)	−1.29	0.1994
SH	2.1 ^ac^	68	1.7 (*n* = 54)	2.4 (*n* = 68)	−2.96	0.0037
Oxidized						
BL1	2.1	51	1.7 (*n* = 71)	2.7 (*n* = 51)	−3.93	0.0001
BL2	2.0	63	1.5 (*n* = 59)	2.5 (*n* = 63)	−3.38	0.0010
CS1	1.8	53	1.3 (*n* = 69)	2.5 (*n* = 53)	−4.91	<0.0001
CS2	2.1	57	1.5 (*n* = 65)	2.7 (*n* = 57)	−4.62	<0.0001
PN	2.0	53	1.5 (*n* = 69)	2.6 (*n* = 53)	−4.72	<0.0001
SH	2.0	50	1.5 (*n* = 72)	2.8 (*n* = 50)	−4.98	<0.0001
Pungent						
BL1	1.2	39	1.0 (*n* = 83)	1.8 (*n* = 39)	−2.83	0.0055
BL2	1.3	47	1.1 (*n* = 75)	1.6 (*n* = 47)	−2.09	0.0385
CS1	1.1	46	0.6 (*n* = 76)	2.0 (*n* = 46)	−5.37	<0.0001
CS2	1.1	38	1.0 (*n* = 83)	1.5 (*n* = 38)	−1.94	0.0550
PN	1.2	39	0.9 (*n* = 83)	1.9 (*n* = 39)	−3.9	0.0002
SH	1.1	40	1.0 (*n* = 82)	1.5 (*n* = 40)	−1.84	0.0687

^1^ The *t*-value and *p*-value represent the result of performing a *t*-test using rating data between the two groups that were selected or not selected for CATA response. ^2^ Shared alphabetical letters mean no significant differences. Mean values without letters show no significant differences between samples.

**Table 4:** Demographic information of consumers.

	All Participants (*n* = 122)		Users (*n* = 77)		Non-Users (*n* = 45)	
	Frequency	(%)	Frequency	(%)	Frequency	(%)
**Sex**						
Male	52	42.6	30	39.0	22	48.9
Female	70	57.4	47	61.0	23	51.1
**Age**						
19–26 years	86	70.5	49	63.6	37	82.3
26–35 years	31	25.4	25	32.5	6	13.3
36–45 years	3	2.5	2	2.6	1	2.2
46–55 years	1	0.8	1	1.3	0	0.0
56–65 years	1	0.8	0	0.0	1	2.2
**Occupation**						
Student	100	82.0	59	76.6	41	91.2
Employed	15	12.3	13	16.9	2	4.4
Others	7	5.7	5	6.5	2	4.4
**Consumption frequency**						
Never drink	45	36.9	0	0.0	45	100.0
Once a month	59	48.4	59	76.6	0	0.0
2–3 times a month	14	11.5	14	18.2	0	0.0
Once a week	3	2.4	3	3.9	0	0.0
2–3 times a week	1	0.8	1	1.3	0	0.0
**Ease of CATA response**						
Not at all	3	2.5	2	2.6	1	2.2
Not really	28	22.9	13	16.9	15	33.3
Neutral	35	28.7	24	31.2	11	24.5
Somewhat	44	36.1	31	40.2	13	28.9
Very much	12	9.8	7	9.1	5	11.1
**Ease of rating response**						
Not at all	5	4.1	3	3.9	2	4.4
Not really	32	26.2	17	22.1	15	33.3
Neutral	45	36.9	32	41.5	13	28.9
Somewhat	35	28.7	24	31.2	11	24.5
Very much	5	4.1	1	1.3	4	8.9
**Boredom of CATA response**						
Not at all	12	9.8	7	9.1	5	11.1
Not really	57	46.7	41	53.2	16	35.6
Neutral	39	32.0	21	27.3	18	40.0
Somewhat	11	9.0	6	7.8	5	11.1
Very much	3	2.5	2	2.6	1	2.2
**Boredom of rating response**						
Not at all	15	12.3	5	6.5	10	22.2
Not really	59	48.4	41	53.2	18	40.0
Neutral	35	28.7	21	27.3	14	31.1
Somewhat	10	8.2	8	10.4	2	4.5
Very much	3	2.4	2	2.6	1	2.2

**Table 5:** Terminology with significant differences between samples using the CATA and rating questionnaires by all consumers, users, and non-users.

	All ^1^	Users		Non-Users	
	No.	No.	Attributes	No.	Attributes
CATA	14	12	asparagus, burnt toast, hay, lemon, medicinal, moldy, natural gas, rubbery, spicy aroma, sulfur dioxide, tar, tropical fruit	4	honey, raisin, strawberry jam, sulfur dioxide
Rating	18	9	black olive, honey, oxidized, rubbery, smoky, spicy aroma, sweaty, tobacco, yogurt	10	berry, burnt toast, butterscotch, cherry, fig, fruits aroma, molasses, prune, strawberry, strawberry jam

^1^ Terms with significant differences by all consumers are shown in Table 2.

**Table 6:** Consumer’s acceptability of wine samples by all consumers, users, non-users and each clusters using total consumers’ liking data ^1,2^.

	Consumer Liking by Familiarity			Consumer Liking by Cluster			
	All (*n* = 122)	Users (*n* = 77)	Non-Users (*n* = 45)	Cluster 1 (*n* = 47)	Cluster 2 (*n* = 36)	Cluster 3 (*n* = 11)	Cluster 4 (*n* = 22)
BL1	5.1 ^ab^	5.3 ^ab^	4.7 ^bc^	5.7 ^a^	3.9 ^abc^	5.1 ^c^	5.2 ^bc^
BL2	4.8 ^bc^	4.8 ^bc^	4.7 ^bc^	5.5 ^ab^	4.3 ^ab^	6.3 ^b^	3.0 ^d^
CS1	5.4 ^a^	5.6 ^a^	4.9 ^ab^	5.2 ^bc^	4.4 ^a^	7.5 ^a^	6.1 ^ab^
CS2	4.5 ^c^	4.7 ^c^	4.2 ^c^	4.8 ^c^	3.3 ^b^	7.1 ^ab^	4.5 ^c^
PN	5.2 ^a^	5.6 ^a^	4.5 ^bc^	5.3 ^abc^	3.6 ^bc^	7.0 ^ab^	6.7 ^a^
SH	5.4 ^a^	5.5 ^a^	5.3 ^a^	5.8 ^a^	4.2 ^ac^	6.8 ^ab^	5.6 ^b^

^1^ Consumer acceptability was evaluated using a nine-point hedonic scale. ^2^ Shared alphabetical letters in the same column means no significant differences.

## Data Availability

The data presented in this study are available on request from the corresponding author. The data are not publicly available due to the institutional data policy.
